# Quantification of fat in the posterior sacroiliac joint region: fat volume is sex and age dependant

**DOI:** 10.1038/s41598-019-51300-y

**Published:** 2019-10-17

**Authors:** Amélie Poilliot, Terence Doyle, Joanna Tomlinson, Ming Zhang, Johann Zwirner, Niels Hammer

**Affiliations:** 1 Department of Anatomy, University of Otago, Dunedin, New Zealand; 2 School of Medicine, University of Otago, Dunedin, New Zealand; 3 Department of Macroscopic and Clinical Anatomy, Medical University of Graz, Graz, Austria; 4 Department of Orthopaedic and Trauma Surgery, University of Leipzig, Leipzig, Germany; 5 Fraunhofer IWU, Dresden, Germany

**Keywords:** Ageing, Bone quality and biomechanics, Skeleton, Ligaments

## Abstract

Fat is appreciated as a structural component of synovial joints. It may serve a shock-absorbing function for the incongruent surfaces, vessels and ligaments, but has not been investigated in the posterior sacroiliac joint (PSIJ). Sixty-six cadaveric hemipelves were serially-sectioned and photographed. The amount of visible fat in the PSIJ was quantified using a modified version of Cavalieri’s method. Total volume, fat volume and fat percentage of the PSIJ were calculated in predefined sub-regions. Fat is consistently present in the PSIJ (1.9 ± 1.3 cm^3^). Fat volume correlates with the PSIJ total volume (p < 0.0001; r = 0.73) and age (p = 0.024; r = 0.24), and is smaller in males (1.4 ± 0.8 cm^3^) than females (2.4 ± 1.5 cm^3^). Fat volumes in the middle and inferior sub-regions of the PSIJ show side- (p < 0.0001) and sex-differences (p = 0.013 females, middle sub-region). Age and PSIJ total volume correlate between sexes in various sub-regions (p = 0.05 females superior sub-region; males inferior sub-region). Fat percentage differs between sexes and sub-regions (p = 0.018 females, superior sub-region) but is independent of age and sides. The presence of fat within the PSIJ is a normal finding and shows sex-dependant and age-related differences. It is unclear whether fat is linked to age-related degeneration or has a shock-absorbing role in stress- and load-dissipation in the PSIJ.

## Introduction

Fatty deposits around a synovial joint may have a shock-absorbing and joint stabilising function^1^. The tissue ‘filled with adipocytes’ within the posterior sacroiliac joint region (PSIJ) is thought to compensate for the incongruent surfaces of the sacrum and ilium posteriorly^2^. The presence of fat in the PSIJ has not been reliably confirmed, and the reasons for its onset and presence are not clear to date. Although some anatomical literature on the sacroiliac joint (SIJ) reports fat^2–10^, there are no studies to date attempting to quantify the fat within the healthy SIJ, nor do studies investigate its potential function within the joint^11^. Knowledge on the presence of fat in the SIJ could provide useful information about the joint physiology, and the clinical and biomechanical implications of its presence.

Several morphology studies indicate the presence of fat within specific layers of the PSIJ ligaments, such as between the meshes of the interosseous ligament (ISL)^3,6,7^, the posterior sacroiliac ligament (PSL)^4^, the sacroiliac part of the iliolumbar ligament^5,10^ and the middle of the long posterior sacroiliac ligament (LPSL)^8,9^, potentially related to nerve supply or vascular feeders^3–10^. Histological findings have described fat being present within the anterior sacroiliac ligament (ASL) in the middle part of the SIJ, with fatty tissue present in the PSIJ region^7^. In addition, studies have described ‘loose connective tissue’ or ‘fat’ surrounding neurovascular structures as they travel through the deep portion of the long posterior sacroiliac ligament in the PSIJ^8,12^. In regards to the potential function of fat within the PSIJ, only one study by Bakland & Hansen comments that the tissue ‘filled with adipocytes’ within the PSIJ diminishes the incongruent surfaces of the sacrum and ilium posteriorly^2^.

Regarding the function of adipose tissue within joints, studies have paid particular attention to articular fat pads within the synovial joints including the knee (infrapatellar fat pad)^1,13–16^ and hip^17–19^. These fatty deposits are said to aid in the neurovascular distribution to neighbouring structures^1^. Reports have discussed the fat pads’ direct involvement in producing pain due to the abundance of substance P fibres and their involvement in the eruption and progression of osteoarthritis^1,13–17,19^. Furthermore, fat has been recognised as having an important endocrine function in its capacity in controlling body homeostasis and would thus have the same function within the joints through articular fat pads^20–22^.

The aim of this cadaveric study was to determine whether adipose tissue within the PSIJ is a common finding. A secondary aim was to quantify the amount of fatty tissue within the SIJs of individuals with no stated pathologies or disorders of the pelvis, serving as a reference for the elderly population.

The hypotheses of this study were (1) fat is consistently present in the PSIJ and is a physiological characteristic, and (2) the amount of fat presence increases with age and there is no difference between sexes and sides.

## Materials and Methods

### Specimens

A total of 66 cadaveric hemipelves were included in this study. Group 1 consisted of digitalised slices of 50 embalmed hemipelves (23 paired, 4 unpaired (3 right, 1 left); 12 males, 15 females, mean age 82 ± 9 years), which were bequested to the University of Leipzig, Germany, and were used in previous studies for different aims^23,24^. Group 2 consisted of 16 paired hemipelves (4 males, 4 females; mean age 86 ± 9 years) acquired through the Univeristy of Otago, Dunedin. These cadavers were embalmed in a supine position using an ethanol-based mixture and had no known history of lower back pain or SIJ-related pathology and no records nor signs (e.g.: scars) upon examination indicating pathology. All body donors while alive gave written and informed consent to the donation of their bodies after their death for teaching and research purposes. The University of Leipzig, Germany granted ethical approval for the use of the tissues for group 1 in conjunction with the Saxonian Death and Funeral Act of 1994 (third section, paragraph 18 item 8). Ethical approval for group 2 was granted by the University of Otago Human Ethics Committee (Health) [ref: H17/20] in conjunction with Māori consultation being sought from the Ngāi Tahu Research Consultation Committee. All methods have been performed according to the relevant guidelines and regulations.

### Slicing and photography

**Figure 1:**
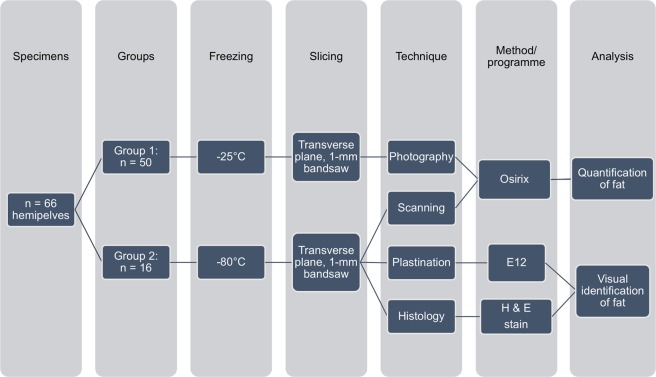
Flow chart of the methodology of the study. E12 is Epoxy resin plastination, H & E is Haematoxilin & Eosin.

Specimens from group 1 were frozen at −25 °C for serial sectioning and then sliced in the transverse plane using a 1-mm band saw (HL-30, Biodur, Heidelberg, Germany), resulting in a series of 14 to 23 slices averaging 5.0 mm in thickness. The superior and inferior aspects of each section were brought to room temperature, and photographed with a digital camera (Lumix FX-9, Panasonic Corp., Osaka, Japan) mounted rigidly on a tripod at a fixed distance^23^. Specimens of group 2 were frozen at −80 °C and sliced in the transverse plane using a 1-mm band saw (B16, Butcher Boy Machines, Selmer, USA), resulting in a series of 17 to 33 slices. The sections obtained averaged 2.25 mm in thickness. The sections were defrosted to room temperature and both surfaces were scanned using a regular scanner between sheets of clear plastic (Perfection V700 Photo scanner, Seiko Epson, Suwa, Nagano, Japan). For an overview of the methods, refer to Fig. 1.

### Plastination and histology

For the purposes of identifying fat and fine detail anatomy on plastinates, one to three slices from each of the 16 hemipelves in group 2 were plastinated with an epoxy resin mixture (E12/E1/AE20 in a ratio of 95:28:20 parts by weight; BIODUR, Heidelberg, Germany)^25,26^. Four further slices from the central PSIJ region of four randomly-selected specimens were assessed histologically. The PSIJ, including the PSL and part of the iliac and sacral bones on either side, was dissected out of a slice, decalcified with ethylenediaminetetraacetic acid for a week, embedded in paraffin, then sliced at 5 μm and stained with Haematoxilin & Eosin (H&E) for microscopic analysis^27^.

### Manual quantification of fat in the PSIJ region

**Figure 2:**
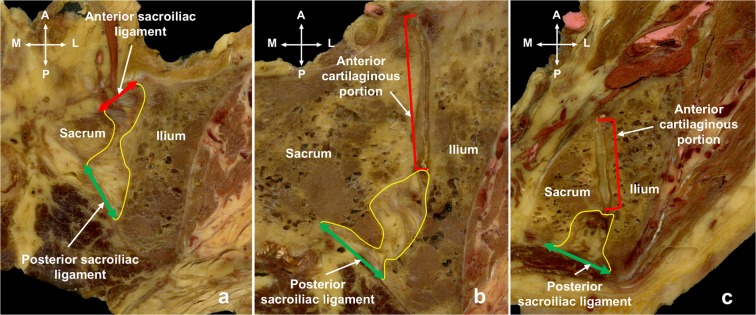
(**a**) A slice from the superior sub-region of the posterior sacroiliac joint. Boundaries of the posterior sacroiliac joint (in yellow) at the level of the first sacral body in horizontal images. Laterally and medially the region is delimitated by the iliac and sacral bones. It is deep to the posterior sacroiliac ligament (green) posteriorly and deep to anterior cartilaginous portion of the sacroiliac joint (red) anteriorly. (**b**) A slice from the middle sub-region of the posterior sacroiliac joint. Boundaries of the posterior sacroiliac joint (in yellow) at the level of the second and third sacral bodies. Laterally and medially the region is delimitated by the iliac and sacral bones. It is deep to the posterior sacroiliac ligament (green) posteriorly and deep to the anterior articular cartilaginous portion (red) anteriorly. (**c**) A slice from the inferior sub-region of the posterior sacroiliac joint. Boundaries of the posterior sacroiliac joint (in yellow) at the level of the third and fourth sacral bodies. Laterally and medially the region is delimitated by the iliac and sacral bones. It is deep to the posterior sacroiliac ligament (green) posteriorly and deep to the anterior articular cartilaginous portion (red) anteriorly. A: anterior, L: lateral, M: medial, P: posterior.

Osirix (version 9.0.2, Pixmeo Sarl 2016, Geneva, Switzerland) was used to quantify the amount of fat within the PSIJ. One author (AP), trained in anatomy, segmented these areas based on anatomical landmarks of the SIJ previously described by Lube *et al*.^28^ and Hammer *et al*.^29^. Boundaries of the PSIJ were defined as follows: laterally the iliac bone, medially the sacral bone, posteriorly the posterior border of the PSL, anteriorly the anterior border of the ASL (Fig. 2a) or the articular cartilage (Fig. 2b,c). Superiorly the superior part of the sacral ala, and inferiorly the last whole slice where the PSL and the ASL visibly merge together. This region of interest (ROI) was circled on each slice to include both sides of all the sections of all specimens. The area of the ROIs was produced by Osirix using a graphpaper scale situated behind the section allowing recalibration of pixels into centimetres. Each specimen was then split into three parts in a horizontal plane: the superior, middle and inferior sub-regions. The superior sub-region was created by the superior third of the slices of the specimen. The other two sub-regions were created using the middle and inferior thirds as shown in Fig. 2.

### Calculation of the posterior sacroiliac joint volume, fat volume and fat percentage

An adapted version of Cavalieri’s method^30,31^ for volume estimation was applied to calculate the volume of the PSIJ using the following formula:

$${{\bf{V}}}_{{\boldsymbol{totalPSIJ}}}={\boldsymbol{d}}({\boldsymbol{\Sigma }}{{\bf{A}}}_{{\boldsymbol{surf}}})$$

Where **V**_**totalPSIJ**_ represents the total volume of the PSIJ in cm^3^, **d** is the distance between each slice (cm) and **A**_**surf**_ is the area of each slice (cm^2^). In this study, both sides of a single slice were used in the the overall calculation of the volume, therefore the average of the distance d was calculated as:

$${\boldsymbol{d}}=\frac{{\boldsymbol{slice}}\,{\boldsymbol{thickness}}+{\boldsymbol{bandsaw}}\,{\boldsymbol{width}}}{2}$$

For group 1, the distance d was 3.0 mm and in group 2, d was 1.60 mm. Within each ROI area, the same author (AP) circled the area perceived to be fat, according to colour and texture. The volume of fat within the PSIJ was calculated using the following formula:

$${{\boldsymbol{V}}}_{{\boldsymbol{fat}}}={\boldsymbol{d}}({\boldsymbol{\Sigma }}{{\boldsymbol{A}}}_{{\boldsymbol{fat}}})$$

Where **V**_**fat**_ is the total volume of fat in the PSIJ (cm^3^), **d** is the distance between each slice (cm), and **A**_**fat**_ (cm^2^) is the area of fat on a slice. Finally, the percentage of fat within the PSIJ was calculated:

$${\boldsymbol{Total}}\,{\boldsymbol{fat}} \% =\frac{{{\boldsymbol{V}}}_{{\boldsymbol{fat}}}}{{{\boldsymbol{V}}}_{{\boldsymbol{totalPSIJ}}}}{\boldsymbol{x}}\,100$$

### Statistical analyses

Graphpad Prism (version 7.0a, San Diego, USA), SPSS 23.0 (IBM, IL, USA) and Microsoft Office (version 16.16.4, Washington, USA) were used for statistical analyses. Data distribution was determined using the D’Agostino and Pearson normality-test. For normally distributed data, side- and sex-comparisons were carried out using a t-test or a one-way ANOVA for multiple assessment of the distribution of fat in the pre-defined superior, middle and inferior sub-regions of the PSIJ. For non-parametric data, a Mann-Whitney U-test was utilised, or a Kruskal-Wallis test with Dunn’s post-hoc correction, for multiple group comparisons of the sub-regions. Age correlations with fat volume, total PSIJ volume, fat volume and fat percentage between sexes and sides were assessed using a two-tailed Spearman-r test for non-parametric data or a one-tailed Pearson-r test for parametric data. A perfect correlation was defined as equal to 1.0, very strong as ≥0.8, moderate as ≥0.6, fair as ≥0.3, and poor ≥0.1 based on Chan *et al*.^32^. P values of 0.05 or less were considered statistically significant. Values are given as means ± standard deviations (s.d.) of the sample.

Intra- and inter-observer reliability was determined based on 20 specimens and assessed by two examiners both trained in anatomy (A.P. and J.T.). Both were blinded to each others’ measurements and the tissue demographics. Using Cronsbachs α and a two-way, mixed intra-class correlation coefficient (ICC), a good result was set at ≥0.70, and ≥0.90 being excellent. Cronbachs α result was 0.98 for the total PSIJ values and 0.97 for fat volume. An ICC result of 0.77 for the total PSIJ volume and 0.83 for the fat volume was found.

## Results

### Fat volume in the PSIJ is larger in females than in males and increases with age

**Figure 3:**
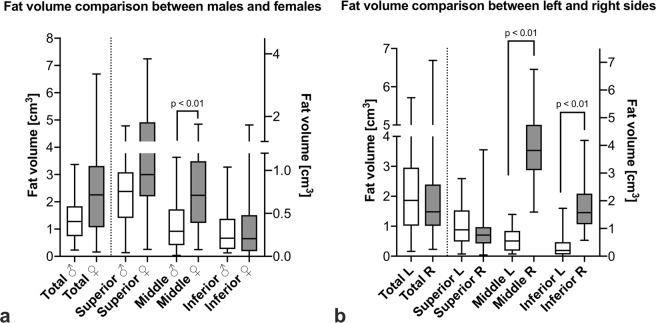
(**a**) Box plot of fat volume distribution between sexes in the total joint and in the superior, middle and inferior sub-regions of the posterior sacroiliac joint. (**b**) Box plot of fat volume distribution between sides in the total joint and in the superior, middle and inferior sub-regions of the posterior sacroiliac joint. The outlines of the boxes indicate the 25% and 75% percentile, the solid horizontal black line the median. The whiskers indicate the minima and maxima. Female: ♀, male: ♂, left: L, right: R.

The fat volume in the PSIJ was lower in males (n = 31; mean 1.4 ± 0.8 cm^3^) than in females (n = 35; mean 2.4 ± 1.5 cm^3^; p ≤ 0.01; Fig. 3a). There were no differences between sides, and there was a poor correlation between age and fat volume (r = 0.24; p = 0.02). There was also a moderate correlation between the fat volume and the total PSIJ volume (r = 0.73; p ≤ 0.01).

Furthermore, the fat volume was significantly different in each sub-region for both sexes pooled (p ≤ 0.01 for all regions), with the superior sub-region containing the highest fat volume (mean 1.0 ± 0.7 cm^3^), followed by the middle (mean 0.6 ± 0.5 cm^3^) and the inferior sub-region (mean 0.3 ± 0.3 cm^3^). Unpooled results showed no statistical difference between the volume of fat in the superior and middle sub-regions in females (p = 0.40) and middle and inferior sub-regions in males (p > 0.99) but was statistically different in the other sub-regions within the same sex (p ≤ 0.01).

When comparing sexes, the middle sub-region of the joint contained more fat in females than in males (p ≤ 0.02) (Fig. 3a). In males, there was a fair correlation between age and the fat volume in the middle (r = 0.33; p ≤ 0.04) and inferior sub-regions of the joint (r = 0.39; p ≤ 0.02); whereas in females, there was only a fair correlation between age and the fat volume in the inferior sub-region (r = 0.30; p = 0.04), no correlation was noted in other sub-regions. In regards to sides, the middle and inferior sub-regions contained more fat in the right side (n = 34; 4.0 ± 1.2 cm^3^ middle sub-region; 1.7 ± 0.8 cm^3^ inferior sub-region; p ≤ 0.01) than the left (n = 32; 0.6 ± 0.4 cm^3^ middle sub-region; 0.4 ± 0.8 cm^3^ inferior sub-region; p ≤ 0.01) (Fig. 3b).

### Total PSIJ volume is larger in females than in males and shows broad inter-individual variation

**Figure 4:**
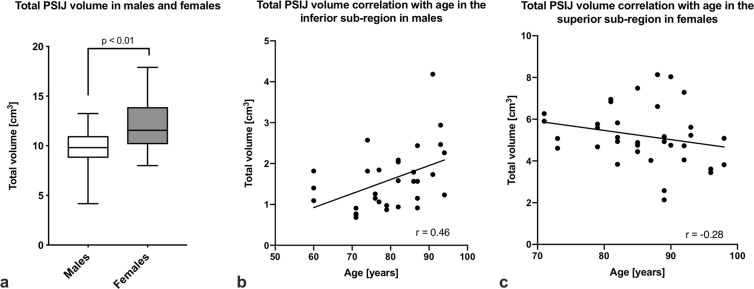
(**a**) Box plot of the total posterior sacroiliac joint (PSIJ) volume between males and females. The outlines of the boxes indicate the 25% and 75% percentile, the solid horizontal black line the median. The whiskers indicate the minima and maxima. Correlations of PSIJ volume with age in the inferior sub-region in males (**b**) and in the superior sub-region in females (**c**).

The total PSIJ volume ranged from 4.2 to 17.9 cm^3^. Males had a smaller PSIJ volume (9.6 ± 2.2 cm^3^) than females (12.0 ± 2.7 cm^3^) (p ≤ 0.01; Fig. 4a). There were no significant differences in total volume between left (11.4 ± 2.9 cm^3^) and right (10.4 ± 2.6 cm^3^) sides (p = 0.15) or any age-related differences (p = 0.21). In both sexes pooled, the superior sub-region has the largest total volume (4.9 ± 1.4 cm^3^), followed by the middle (3.9 ± 1.5 cm^3^) and the inferior sub-region (1.8 ± 0.9 cm^3^). When the sexes were unpooled, the volumes of all sub-regions were significantly different (p ≤ 0.01) except between the superior and middle sub-regions in females (p = 0.81). There was a fair negative correlation between age and the total PSIJ volume in the superior sub-region in females (r = −0.28; p ≤ 0.05), when in males this was a very strong positive age-correlation in the inferior sub-region (r = 0.46; p ≤ 0.01; Fig. 4b,c).

### Fat percentage is greater in the PSIJ of females

Fat percentage showed no side dependency nor correlation with age with both sexes pooled. In all sub-regions, there was a higher fat percentage in the superior sub-region in females than in males (p ≤ 0.02). In females, there was a fair correlation between age and the fat percentage in the inferior sub-region of the joint (r = 0.39; p ≤ 0.01) when in males, there was no age-correlation in any of the sub-regions of the joint.

### Plastination and histology reveals fat between the meshes of ligamentous tissue

**Figure 5:**
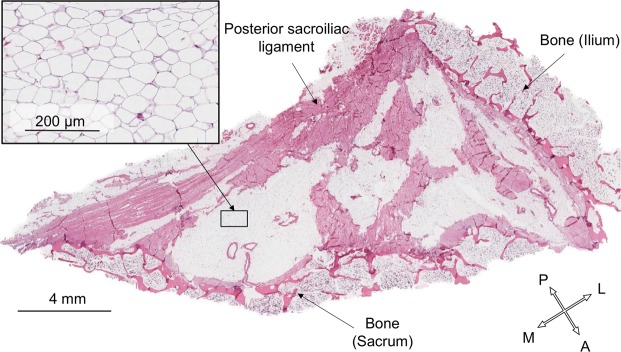
Histological section of the posterior sacroiliac joint region of a 71 year-old female. The black box represents the close-up region showing adipose cells. A: anterior, L: lateral, M: medial, P: posterior.

The E12-plastinated slices showed transparent, yellowish areas, which appears to be fat, between the meshes of ligaments, which, in contrast, were darker streaky structures of brownish or reddish colour^25,33,34^. In microscopy, adipose tissue was found within the PSIJ. This showed a ‘signet ring’ appearance, visual as empty compartments with only a thin rim of cytoplasm and nuclei along the edge^27,35^. In the four specimens, fat tissue cell membranes were clearly visible between the ligamentous tissue in the PSIJ (Fig. 5).

## Discussion

This study confirms that fat tissue reliably exists within the PSIJ region of the elderly and, for the first time, quantifies the amount of fat within the PSIJ compartment. As seen in other joints, fat contributes to joint biomechanics as a shock absorbing structure^1,13–19,36^. The kinematics of the SIJ have been reported to be influenced by a number of factors, involving sex-related morphology, age, body weight, side/leg asymmetry, as well as ligament and muscle imbalance^37–44^. Between sexes, biomechanics of the SIJ are different where females experience higher stresses, loads and ligament strains distributed over larger contact areas around the SIJ compared to males. This has been attributed to differences in SIJ size, its surrounding ligaments and in a different center of gravity between the sexes^37^. This study found that a greater volume of fat was present in females compared to males, as well as larger overall tissue volumes in the PSIJ area, indicating sex-dependent differences. Differences according to sides were found in the sub-regions of the PSIJ. Also an age-related increase in fat volume was noted in both sexes, accompanied by increasing values of fat percentage in females. This suggests that fat content may be a variable that may contribute to the biomechanical differences between sexes or just be an indicator of such a difference.

### Fat within the PSIJ is likely to be a normal component of the healthy joint

Fat may potentially serve as a cushion for surrounding hard tissues, making the PSIJ more rigid when compressed, thus allowing the SIJ ligaments to become preloaded, in order to effectively allow stress distribution in an uncrimped state of collagens. Therefore, the first hypothesis can be accepted, stating that fat in the PSIJ may likely form a physiological element to the joint. But, as stated for the plantar fat pad, fat has a direct correlation to weight in overweight individuals^45^, which may need to be explored in this region. Past studies have found that plantar fat pad size has a direct correlation with body height, suggesting that fat development may also be related to skeletal size^18^. This hypothesis could not be investigated in this study, as there was a limited number of cadavers available in an anatomically fixed condition involving ethanol. Furthermore, fat aids in the neurovascular distribution to neighbouring structures^1,7,8,46^. A network of neurovascular bundles including the posterior rami of spinal segments and the blood supply to the joint travel within the fat of the PSIJ supplying the LPSL^7,9^. The superior sub-region was reported to contain more fat in both sexes. This fat could be related to the larger neurovascular structures passing both anteriorly and posteriorly at the lumbosacral junction. These include the lumbosacral cord and the vasculature of the anterior pelvis^11,47,48^.

The biomechanics of the SIJ can alter with age and weight gain. It has been reported that the overall joint mobility decreases with age, and is sometimes obliterated completely because the joint can erode significantly and may even fuse in the elderly^3,11,38–41,49^. In obese individuals, the morphology of the SIJ is affected. Their SIJs have a less uniform joint space with a increased ill-defined subchondral sclerosis, which would contribute to SIJ pain^44^. However, because the body mass index (BMI) of the individuals in this study were unknown, it was not possible to consider the impact of BMI on fat volume within the PSIJ. Further research should investigate this variable to understand its potential influence on altered loads and stresses on the joint. Furthermore, future studies should assess the morphological aspect of the anterior auricular portion of the SIJ as it may be related to SIJ biomechanical loading, stress distribution and potentially fat presence in the posterior portion especially if it has an age-related influence.

In regards to sex-related fat content of the PSIJ, the second hypothesis was rejected as females had a higher fat content in the middle sub-region of the joint and age correlated with an increase in fat content in the inferior sub-region and both the middle and inferior sub-regions in males. In fact, females also had, in general, a higher fat percentage than males, suggesting that females have more fat regardless of their joint size. If fat has a load-bearing function it may compensate for the higher forces and loads subdued by females during life due to their smaller articular joint surfaces^37,50^. Furthermore, studies have shown that there is an increase in stress in the middle and inferior sub-regions of the subchondral bone plate and interosseous region when walking, suggesting that the joint needs more ‘cushion’ for this increase in loads in those sub-regions^51,52^. Fatty infiltration in response to degeneration has been observed in cases of sarcopenia, and may have similar effects in the PSIJ from a ligamentous perspective^53–55^. Additionally, fatty infiltration is common in response to inflammation or trauma to ligamentous structures, where fat is an intermediate step during the reparation of structures^56^. In this study, there is no proof on whether the fat content is a result of ‘infiltration’ within the joint as it is present in all individuals. Even though it increases with age, fat may simply increase over time rather than abnormally infiltrate the joint. A specific study on a younger cohort should be sought to ilucidate this hypothesis.

The correlation between PSIJ fat volume and total PSIJ volume suggests a proportionality between the fat content and the size of the joint space. This supports the hypothesis that fat forms as a natural physiological and functional element as it is directly related to PSIJ volume. Total fat volume between sides was not different, however, there was a significant difference when specifically looking at the results of unpooled sub-regions of the PSIJ, where generally the right side contained more fat compared to the left side. Studies have shown that the right leg is often the dominant limb, this may have an influence on morphology of the tendons and ligamentous structures^57–59^ and potentially an impact on the ligament-fat ratio. Hence, the middle and inferior sub-regions of the PSIJ of the dominant leg may contain more fat to compensate for the increase in forces on the joint in response to an increase in ligament strain in that area. Furthermore, leg length discrepancies (≥2 cm) have shown that the load imbalance may increase the possibility of having a painful SIJ, which may suggest that fat may be present to ‘balance out’ the unequal loads and protect neurovascular structures^42^. However, it is unknown what limb was dominant in the specimens of this study.

### Sex-dependent differences in PSIJ volumes are indicative of sexual dimorphism

We have determined that the PSIJ has a larger volume in females than in males. Sexual dimorphism of the SIJ is well recognised as appearing in both the anterior articular and posterior interosseous parts of the SIJ^60,61^. Mechanisms involved in the adaptation of female pelves in pregnancy and childbirth may contribute to the composition of the SIJ ligaments and pubic symphysis^49,59,61–63^. It is however unclear whether the here-observed sex-dependent features are the effects of pregnancy, sex-specific findings, or a combination of the two. Steinke *et al*.^23^ have shown that females also had larger ISL volumes than males. This is supported by the results of this study as the ISL fills the PSIJ. Joukar *et al*.^37^ determined that females are subjected to higher loads than males because of their smaller articular joint surfaces. These smaller articular surfaces result in a more mobile SIJ, causing larger peak stresses to the joint which would explain the larger ISL volumes in females as compensatory mechanisms^37,50^. In this regard, fat within the SIJ may serve as protection to the neurovascular structures to prevent damage resulting from a decreased joint space.

**Figure 6:**
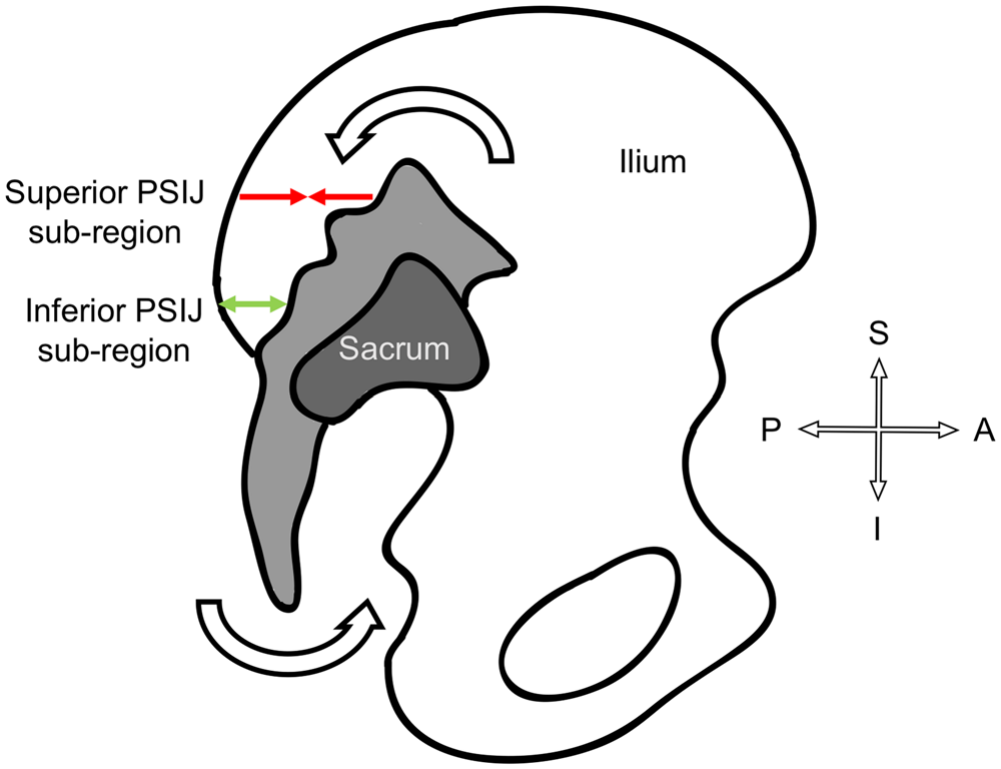
Sagittal view of a left coxal bone with a complete sacrum showing the possible effect of age-related counternutation of the sacrum to the posterior sacroiliac joint region (PSIJ). The superior sub-region (red) narrows whilst the inferior sub-region (green) widens.

Age-related change of the anterior aspect of the SIJ has been investigated previously^38–41^. One of the factors of degeneration and a clinical sign of osteoarthritis is ‘joint space narrowing’, which may also be a feature applicable to the PSIJ region^38–40,64^. In both sexes, the volume of the superior and inferior sub-regions changed with age, where the inferior sub-region gets larger in males and the superior sub-region smaller in females. This feature may be a natural physiological occurrence in both sexes resulting from weight gain; however, BMI was unknown in this cohort. For the females, this may form a post-menopausal response as menopause significantly affects bone density which alters the biomechanics of the lumbar spine and could result in a slight caudal displacement of the sacrum^65–69^. This may alter the forces running through the SIJ which needs more ‘cushioning’ in response to the increase in stress to the joint. In fact, this finding might be a common degenerative factor of the joint, where, with age, the superior sub-regions of the joint narrows, which results in a broadening of the inferior sub-region due to a slight counternutation of the sacrum (Fig. 6). The joint cartilage erodes and the anterior aspect of the joint sometimes fuses and narrows which is a common phenomenon for people over 60 years old of age^49,70^. Significant results were only found in the inferior sub-region in males and the superior sub-region in females, which may be because of the small sample size. In fact, a larger sample size may reflect that the superior sub-region narrows and the inferior sub-region broadens in both sexes.

All specimens included in this study were older than 60 years of age and the total sample size was limited. Our results lack population validity and investigations on younger cohorts are required to verify the finding that age impacts fat presence within the PSIJ, or if the age-related increase in fat in the PSIJ only exists in a geriatric population. Furthermore, any history of pelvic pathology or pain is unknown in the underlying samples, which may form solid grounds for considering this a cross-sectional sample instead of a healthy sample with a higher likelihood of age-related degeneration. In addition, ethanol embalmed cadavers were used in this study. This technique may potentially have removed a part of the lipid phase from tissues, which suggests that the estimated values cannot be applied to other embalmed, fresh or live tissues. Stereological analysis of fresh tissue would be ideal to quantify fat within the PSIJ as it would be able to quantify smaller quantities of fat that may not be visible using the Osirix method.

Limitations to the manual segmentation method include the potential bias introduced by plain photography and the resolution of the images, which may have caused partial volume effects. These limitations in the images, however, may have only played a minor role, as this would only apply to boundary areas of the fat. As fat was entirely assessed on colour and texture appearance, this may bring a subjective element to the study. Here, however, high intra- and inter-rater reliabilities speak in favour of the method, suggesting this method may be applied to other joints or anatomical regions. Further errors may have been introduced by minute deviations when slicing the tissues. Ultimately, it must be emphasised that although this study aimed to quantify the fat in the PSIJ, these methods only result in an estimation of the amount of fat and total PSIJ volumes.

## Conclusions

This study has demonstrated the consistent presence of adipose tissue within the PSIJ region. Fat within the PSIJ is sex-dependant and age-related and is thought to be linked with natural degeneration. The fat may have a shock-absorbing role for during PSIJ compression in the different sub-regions. The novel method presented appears to be reliable and may be applied to other joints or anatomical regions.

## Data Availability

The data acquired in the course of this study are available from the corresponding author on request.
